# Data-Driven Investigation of Gait Patterns in Individuals Affected by Normal Pressure Hydrocephalus

**DOI:** 10.3390/s21196451

**Published:** 2021-09-27

**Authors:** Kiran Kuruvithadam, Marcel Menner, William R. Taylor, Melanie N. Zeilinger, Lennart Stieglitz, Marianne Schmid Daners

**Affiliations:** 1 Product Development Group Zurich, Department of Mechanical and Process Engineering, ETH Zurich, 8092 Zurich, Switzerland; kiranku@ethz.ch; 2 Institute for Dynamic Systems and Control, ETH Zurich, 8092 Zurich, Switzerland; menner@ieee.org (M.M.); mzeilinger@ethz.ch (M.N.Z.); 3 Laboratory for Movement Biomechanics, Institute for Biomechanics, ETH Zurich, 8093 Zurich, Switzerland; bt@ethz.ch; 4 Department of Neurosurgery, University Hospital Zurich, 8091 Zurich, Switzerland; lennart.stieglitz@usz.ch

**Keywords:** hydrocephalus, gait analysis, kinematic measurement, machine learning, neural network, regression analysis, wearable sensors

## Abstract

Normal pressure hydrocephalus (NPH) is a chronic and progressive disease that affects predominantly elderly subjects. The most prevalent symptoms are gait disorders, generally determined by visual observation or measurements taken in complex laboratory environments. However, controlled testing environments can have a significant influence on the way subjects walk and hinder the identification of natural walking characteristics. The study aimed to investigate the differences in walking patterns between a controlled environment (10 m walking test) and real-world environment (72 h recording) based on measurements taken via a wearable gait assessment device. We tested whether real-world environment measurements can be beneficial for the identification of gait disorders by performing a comparison of patients’ gait parameters with an aged-matched control group in both environments. Subsequently, we implemented four machine learning classifiers to inspect the individual strides’ profiles. Our results on twenty young subjects, twenty elderly subjects and twelve NPH patients indicate that patients exhibited a considerable difference between the two environments, in particular gait speed (*p*-value p=0.0073), stride length (*p*-value p=0.0073), foot clearance (*p*-value p=0.0117) and swing/stance ratio (*p*-value p=0.0098). Importantly, measurements taken in real-world environments yield a better discrimination of NPH patients compared to the controlled setting. Finally, the use of stride classifiers provides promise in the identification of strides affected by motion disorders.

## 1. Introduction

Gait patterns can provide a good insight into the overall health status of a subject, since walking involves well-coordinated participation of different body systems [1]. Current methods rely on the evaluation of walking characteristics in controlled laboratory environments, which are likely to falter the observations because of psychological pressure and resulting bias [2,3]. Here, in-depth studies can support our understanding of how the environment can affect measurements of subjects of different age groups and health status, as it was the case in a previous pilot study by Renggli et al. [4], which has demonstrated how a controlled lab setting is able to affect healthy elderly subjects, resulting in a significant deviation from their natural gait patterns. By virtue of these observations, the current study hypothesizes that such deviations in gait characteristics between real-world and controlled lab environments could also be expected for subjects that suffer from neurological motor disorders, and that this difference could be of vital importance in the detection and diagnosis of neurological disorders.

Normal pressure hydrocephalus (NPH) is a chronic and progressive condition that affects predominantly elderly subjects. It is associated with the accumulation of excess cerebrospinal fluid in the brain’s ventricles and is characterized by cognitive deterioration, urinary incontinence and gait disturbance [5]. While the condition is treatable through surgical diversion of the cerebrospinal fluid (accomplished with the implantation of a draining shunt) [6], only some 10% of all patients are correctly identified, and therefore improvements in diagnostics are needed [7]. The most prominent symptoms of NPH have been shown to be gait disorders [6,8,9], usually determined by visual observation or a direct comparison of gait features (such as stride length or gait speed) between patients and healthy subject groups. Although other studies have investigated the relevance of gait parameters in recognizing gait disorders in hydrocephalus patients [5,10,11,12], to the best of our knowledge, no current study has tried to implement computational methods, such as support vector machines (SVMs) or neural networks, to recognize high-risk NPH subjects based on gait analysis in the real-world environment. Other recent studies integrated deep-learning techniques either for video-based gait analysis of NPH patients in a controlled environment [13] or to predict fall-risk subjects affected by different neurological diseases using recurrent neural networks [14]. While both works show great potential for deep-learning techniques in the field, there is still a lack of studies performed in prolonged sessions in real-world settings.

Currently, the only viable instrumentation that allows for prolonged home monitoring of gait patterns is a wearable system, able to perform measurements of the kinematics outside the confinements of a laboratory and without getting in the way of daily activities [1]. The current study achieves prolonged gait monitoring by using wearable ZurichMOVE www.zurichmove.com (accessed on 22 September 2021) inertial measurement units (IMUs) and the gait extraction algorithms developed by Renggli et al. [4].

The aim of this study was threefold: (1) extend the investigation of the environmental influence on gait patterns to NPH patients by comparing gait parameters obtained during IMU-based gait analysis in both controlled and real-world settings, (2) investigate which of the two testing environments provides more useful diagnostic information by assessing the ability of data captured in each environment to discriminate patients from healthy subjects through statistical tests and logistic regression, and (3) provide an insight into different gait classification approaches (based on SVM and neural networks), which analyse either the time series representation or parametric representation of strides.

## 2. Materials and Methods

### 2.1. Sensors

Measurements have been taken using five ZurichMOVE 9 degrees-of-freedom (DoFs) IMUs. Here an IMU was attached to each ankle and each wrist as well as the chest, using kinesiology tape. The sensors feature full-scale ranges for both accelerometer (±2 g, ±4 g, ±8 g, ±16 g) and gyroscope (±250, ±500, ±1 k, ±2 k ${}^{\circ }$/s) and data are sampled at 50 Hz and logged onto local memory for later downloading and post-processing.

### 2.2. Subjects

This study was approved by the Cantonal Ethics Committee Zurich (BASEC-No. 2018-00051) and Swissmedic (102597735), and all subjects provided their written, informed consent prior to participation. Groups were composed of twenty healthy young subjects (10 male and 10 female, aged 24.9 ± 2.7 years, height 173.3 ± 9.5 cm and weight 68.3 ± 12.7 kg), twenty healthy elderly subjects (10 male and 10 female, aged 75 ± 8.4 years, height 171.4 ± 9.7 cm and weight 70.7 ± 12.1 kg), and twelve NPH patients (5 male and 7 female, aged 76 ± 5.4 years, height 168.4 ± 12.2 cm and weight 75.6 ± 12.6 kg).

### 2.3. Protocol

After calibration, including two five-meter walking trials each at different prescribed foot spacing, all subjects underwent gait evaluation in the controlled lab environment. Gait analysis then involved a 10-m walk, repeated four times (with 180${}^{\circ }$ turnarounds in-between) at the subject’s own preferred speed. Subjects were then requested to continue wearing the five sensors for three further days to record movement patterns in their real-world settings. The measurements protocol is described in detail by Renggli et al. [4].

### 2.4. Gait Analysis

In addition to standard parameters such as stride length and gait speed, a number of additional parameters have been considered (all listed in Table 1), which are specifically indicative or symptomatic for NPH: these include step width, outward rotation of the foot, foot clearance during swing, swing velocity and amplitude of arm movement [5,6,10,12].

In order to extract gait parameters, all IMU data were firstly processed using a stride detection algorithm, based on a technique proposed in a previous study by Renggli et al. [4]. Here, the detection of strides is achieved through an initial peak event detection-based method where walking events such as heel-strike (HS), flat-foot (FF) and toe-off (TO) are identified and labeled. The data series is then segmented by defining the flat-foot event as the start and end points of a stride, and by verifying the sequence FF-TO-HS-FF for every stride candidate. Further empirically determined boundary conditions and a template matching approach, based on dynamic time warping (DTW), are then used to refine the search and obtain the stride candidates.

After the stride events were detected, gait parameters were extracted for each stride. Temporal parameters were obtained by directly processing the IMUs’ data in the local coordinate frame. Spatial parameters on the other hand are extracted by first estimating the orientation of the sensors and then expressing accelerations in a global coordinate frame. Integration of kinematic data makes use of the assumptions that accelerations and velocities at both start and end mid-stances are equal to zero, and that integration errors (caused by drift and errors in the orientation estimation) accumulate linearly throughout the stride and therefore can be easily be accounted for [15,16]. For more details about the parameter extraction algorithm and validation of the system, the reader is referred to [4] (the step-detection algorithm is available at http://doi.org/10.5905/ethz-1007-243, accessed on 22 September 2021).

### 2.5. Statistical Analysis

All statistical analyses were performed using the statistics and machine learning toolbox MATLAB R2019a (The Mathworks Inc., Natick, MA, USA) [17].

In order to compute the extent of differences in walking patterns between controlled and real-world environments in the different subject groups, we compared the median values of the intra-group gait parameters from both environments and tested for significance using a non-parametric Wilcoxon signed rank test (MATLAB functions ‘signrank’), appropriate for small sample sizes and when the normal distribution assumption is not tenable [18]. Resulting *p*-values were corrected for false discovery using the Benjamini–Hochberg correction. These comparisons were performed for all three subject groups: the young healthy control group (YHC), the elderly healthy control group (EHC) and the NPH patients group.

In a second step, we compared the walking patterns of EHC and NPH subject groups for both controlled and real-world environments by means of the Mann–Whitney U-test and a logistic regression-based classifier.

The Mann–Whitney U-test was used to evaluate the null-hypothesis by comparing the median values of the gait parameters of all subjects belonging to EHC and NPH (MATLAB function ‘ranksum’).

For the logistic regression classifier (LRC), the set of median values of the gait parameters for each subject were classified either as EHC or NPH using logistic regression (MATLAB functions ‘templateLinear’ and ‘fitcecoc’), as characterized by cross-entropy loss function and enhanced through lasso regularization as described in Equation (1):

(1) $$J\left(\beta \right)=-\frac{1}{m}\sum_{i=1}^{m}y^{i}\ln\left(h_{\beta }\left(x^{i}\right)\right)+\left(1-y^{i}\right)\ln\left(1-h_{\beta }\left(x^{i}\right)\right)+\frac{\lambda }{2m}\sum_{n}^{j=1}\left|\beta^{j}\right|$$

where J(β) is the cost function, β is a vector of *n* coefficients, $x^{i}$ is a vector of *n* predictor variables that represents the *i*th observation, $y^{i}$ is the true class of the *i*th observation, *m* is the total number of observations, λ is the hyper-parameter of the l1-norm regularization and $h_{\beta }$ is the classifier function:

(2) $$h_{\beta }\left(x^{i}\right))=\frac{1}{1+e^{-(\beta^{T}x^{i}+b)}}$$

with *b* being a scalar bias estimated during optimization. The cost function is optimized through a stochastic gradient descent [17], and the hyper-parameter optimization uses 8-fold cross validation. The performance of the LRC was evaluated through repeated random sub-sampling validation (Monte Carlo cross validation, using 60 iterations).

For all statistical calculations, the significance alpha-level was set to p<0.05.

### 2.6. Data-Driven Approaches for Stride Classification

Four classification techniques were applied for the classification of stride-type of elderly healthy controls and NPH patients and considered only real-world setting measurements. The first classifier was based on a support vector machine (SVM, MATLAB functions ‘templateLinear’ and ‘fitcecoc’), for which a stride profile was determined for each subject. Every measured stride was characterized by the 15 extracted gait parameters and labeled according to the corresponding subject group. According to the stride profile, strides were classified as belonging to either EHC or NPH patients. The classifier is characterized by an l1-norm soft-margin formulation, which makes use of relaxed linear separability. As a result, the cost function J(β) was described using the following equation:

(3) $$J\left(\beta \right)=\gamma \sum_{i=1}^{m}\max\left\{0,1-y^{i}\left(\beta^{T}x^{i}+b\right)\right\}+\sum_{j=1}^{n}\left|\beta_{j}\right|$$

where β is a vector of *n* coefficients, *b* is a scalar bias estimated during optimization, $x^{i}$ is a vector of *n* predictor variables that represents the *i*th observation, $y^{i}$ is the true class of the *i*th observation, *m* is the total number of observations and γ is the slack penalty hyper-parameter for the Hinge loss term [17]. The cost function was optimized through stochastic gradient descent, and 5-fold cross-validation was performed for the optimization of the hyper-parameter.

The second classifier NN-stride was based on a neural network (NN) architecture. Analogously to the SVM-stride approach, the classifier labels every stride according to the group of the corresponding subject by using the 15 extracted gait parameters as the predictor variables. Strides were classified as belonging to either EHC or NPH patients. The architecture of the NN-stride classifier (MATLAB function ‘feedforwardnet’) follows a two-hidden layers design (with 30 and 20 hidden neurons, respectively) associated with a tangent sigmoid activation function. Mean square error was defined as the performance function for the optimization of weights (w), biases (*b*) and the model’s overall learning process, as described in Equation (4):

(4) $$J\left(w,b\right)=\frac{1}{m}\sum_{i=1}^{m}{\left(y^{i}-a^{i}\left(w,b\right)\right)}^{2}$$

where $a^{i}$ are the outputs of the NN. Optimization was achieved through scaled conjugate gradient descent back-propagation [19].

**Figure 1:**
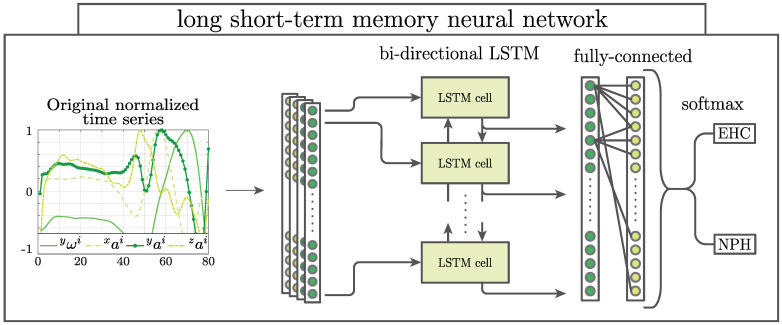
The long short-term memory (LSTM) classifier takes as input a four channel signal composed of the angular velocity around the ankle’s mediolateral axis ${}^{y}\omega^{i}$ and the three linear accelerations ${}^{x}a^{i}$, ${}^{y}a^{i}$ and ${}^{z}a^{i}$ during one stride. The first LSTM cell uses the initial state of the network and the first time sample to update the current cell state and compute the output. Subsequent time samples are fed into the corresponding LSTM cell, which makes use of previous cell states to learn about long and short-time dependencies within and between the signal channels.

A further two classifiers were based on long-short term memory (LSTM) architecture and convolutional neural network (CNN), implemented using the MATLAB 2019a Deep Learning Toolbox [19]. As opposed to the already described methods (SVM-stride and NN-stride) that rely on gait-feature extraction, LSTM and CNN make direct use of the filtered time series of the three-axis accelerometer as well as the time series of the gyroscope, which was aligned with the ankle’s mediolateral axis (to ensure efficiency of the computations, all gait strides were resampled and normalized to 80 time points, as this was the maximum number of data samples captured within any single stride, which therefore avoided downsampling). Consequently, every stride was represented by a four-dimensional time-sequence and labeled as belonging to either EHC or NPH patients. The LSTM classifier (Figure 1) is based on LSTM networks, which are particularly suitable for sequence prediction and classification due to their ability to learn the interplay of complex temporal dynamics and long-term dependencies within the time-sequences [20]. The LSTM model is composed of a bi-directional LSTM layer, a fully connected layer, a softmax layer, and a classification layer that computes the cross-entropy loss. Optimization of the LSTM cell parameters was achieved using the Adam optimizer [19].

**Figure 2:**
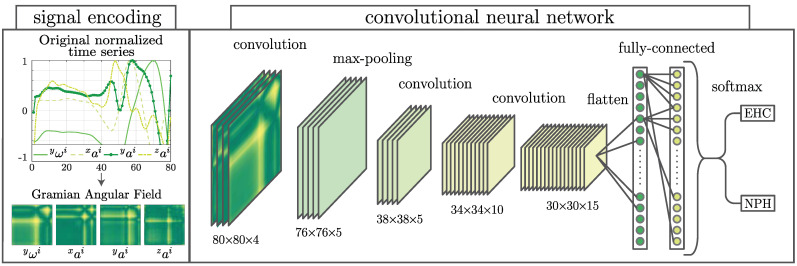
The convolutional neural network (CNN) classifier makes use of a signal encoder, which translates the angular velocity around the ankle’s mediolateral axis ${}^{y}\omega^{i}$ and the three linear accelerations ${}^{x}a^{i}$, ${}^{y}a^{i}$ and ${}^{z}a^{i}$ into a four channel image. This image, which represents one subject’s stride, is then fed into a 3D CNN.

The CNN classifier (Figure 2) is a type of feedforward neural network, often used in image-classification for its feature recognition and extraction capabilities. For this reason, the four-dimensional time series data that represent each stride have been encoded onto 4-channel pictures using a Gramian angular field encoder:

(5) kImi=cos(ϕ1+ϕ1)cos(ϕ1+ϕ2)…cos(ϕ1+ϕn)cos(ϕ2+ϕ1)⋮⋱⋮cos(ϕn+ϕ1)…cos(ϕn+ϕn)

where kImi represents the *k*th channel of an 80×80 pixel image associated to the *i*th stride. The arguments $\phi_{1}\ldots \phi_{n}$ are defined as

(6) $$\phi_{j}=\arccos\left(u_{j}\right)$$

where u is the time series being encoded, which for our purposes correspond to the data series measured by the kinematic sensor ${}^{x}a^{i}$, ${}^{y}a^{i}$, ${}^{z}a^{i}$ and ${}^{y}\omega^{i}$. The CNN classifier is composed of three convolutional layers associated with the rectified linear units activation function (ReLU), a softmax layer, and a classification layer that computes the cross entropy loss. Optimization of the convolutional layers’ weights and biases is achieved through an Adam optimizer (see [19] for details).

We adopted accuracy and the correct detection rate (CDR) as performance metrics of the different machine learning models. Given the number of true EHC ($T_{\mathrm{EHC}}$), true NPH patients ($T_{\mathrm{NPH}}$), false EHC ($F_{\mathrm{EHC}}$) and false NPH patients ($F_{\mathrm{NPH}}$), accuracy and CDR are defined as:

(7) $$\mathrm{Accuracy}=\frac{T_{\mathrm{EHC}}+T_{\mathrm{NPH}}}{T_{\mathrm{EHC}}+T_{\mathrm{NPH}}+F_{\mathrm{EHC}}+F_{\mathrm{NPH}}}$$

(8) $${\mathrm{CDR}}_{\mathrm{EHC}}=\frac{T_{\mathrm{EHC}}}{T_{\mathrm{EHC}}+F_{\mathrm{NPH}}}$$

(9) $${\mathrm{CDR}}_{\mathrm{NPH}}=\frac{T_{\mathrm{NPH}}}{T_{\mathrm{NPH}}+F_{\mathrm{EHC}}}$$

Basing performance solely on accuracy can, however, be misleading, since it does not reflect classification biases or imbalance in the data sets [21]. For this reason, we also extracted receiver operating characteristic (ROC) curves, which capture the robustness of the classifier by plotting the false positive rate to the true positive rate for all possible prediction thresholds, and we derived the area under the ROC curve (AUROC) value for each classifier. All four classification techniques were implemented and compared for their ability to classify the strides. To this end, the performance of each classifier was evaluated using Monte Carlo cross validation.

## 3. Results

### 3.1. Real-World versus Controlled Environment

**Figure 3:**
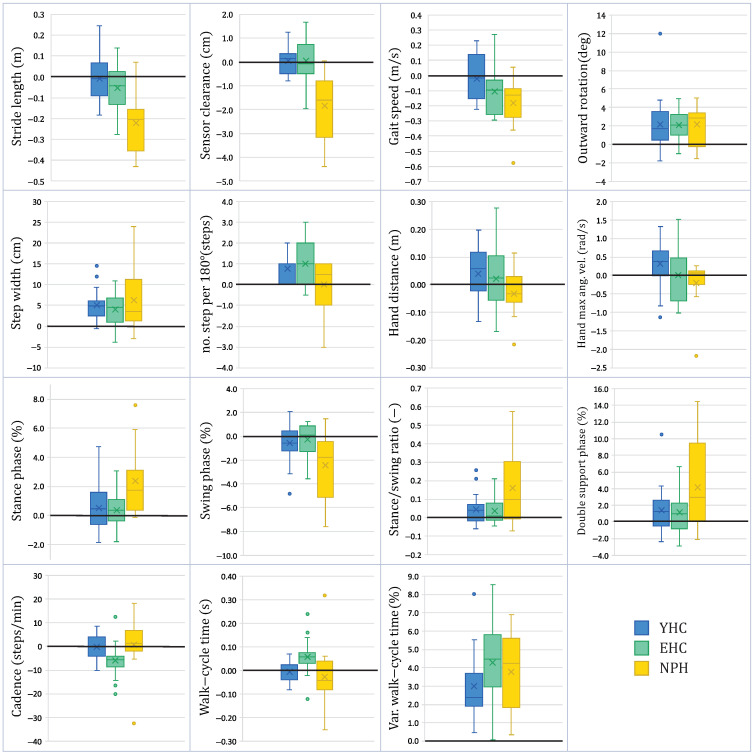
The boxplots illustrate the comparison between controlled and real-world environments for 20 young healthy control (YHC), 20 elderly healthy control (EHC) and 12 normal pressure hydrocephalus patients (NPH). Each plot indicates the absolute difference between the parameter’s median values in each scenario of every subject (${\mathrm{Parameter}}_{\mathrm{real}-\mathrm{world}}^{\mathrm{subject}}-{\mathrm{Parameter}}_{\mathrm{lab}}^{\mathrm{subject}}$). Within each box-plot, the group’s median and mean values are indicated as a line and a cross, respectively, and outliers are indicated as dots. The upper and lower edges of the box span the interquartile range, and whiskers indicate the most extreme data points not considered outliers.

The difference in gait characteristics between the lab and real-world settings is summarized in Figure 3, and statistical results are listed in Table 1. Based on the results from the statistical tests, gait parameters were divided into three clusters: (1) significantly different for all three subjects groups YHC, EHC, NPH; (2) significantly different only for EHC and NPH groups; (3) significantly different only for the NPH group.

#### 3.1.1. Significant Differences between Environments for All Subjects Groups YHC, EHC and NPH

When comparing measurements taken in the real-world setting with the controlled environment, all subject groups exhibited an increase in outward rotation (YHC: 19%; EHC: 16%; NPH: 17%), an increase in step width (YHC: 32%; EHC: 18%; NPH: 23%) and higher variability in the stride time (YHC: 51%; EHC: 58%; NPH: 43%).

#### 3.1.2. Significant Differences between Environments Only for EHC and NPH

In the real-world setting, both EHC and NPH groups show a decrease in gait speed (EHC: 12%; NPH: 44%) compared to the lab environment.

#### 3.1.3. Significant Differences between Environments Only for the NPH Group

Several parameters showed a significant difference only for the NPH group. Compared to lab settings, NPH subjects tended to have a considerable decrease in stride length (4%), a lower sensor clearance (25%), an increased stance/swing ratio (9%), an increase in double support phase (17%), a longer stance phase (4%), and a shorter swing phase (8%) in real-world environments.

The remaining parameters either did not differ significantly for any of the subject groups (in all three groups, traveled hand distance shows only a slight decrease in the real world measurements) or differed exclusively between the healthy subject groups (YHC and EHC). This was the case for the hand’s maximum angular velocity, which exhibited a statistically significant increase only for younger subjects (YHC: 7%), and two remaining parameters, which differed significantly only for EHC subjects: cadence (decrease of 6%) and stride time (increase of 5%).

### 3.2. Elderly Subjects versus NPH Patients

In the comparison between gait patterns of EHC and NPH subjects, outward rotation and stride width are the only parameters that did not achieve significance in either setting (Table 2). In real-world settings, most gait features showed a p<0.0001, and there are only three gait parameters (cadence, stride time, and variability of stride time) that reached the significance level solely in the controlled lab environment.

The logistic regression classifier extended the investigation by using the gait parameters to classify the subjects as either healthy elderly or NPH patient. The confusion matrices in Table 3 show the results for controlled (left) and real-world (right) settings measurements and the CDRs (represented by the diagonal terms of the 2×2 matrices in Table 3) are consistently higher in the real-world environment, with 95.8% of EHC and 91.6% of NPH patients correctly classified, compared to 89.5% and 75.6% for the lab measurements.

### 3.3. Stride Classification through Machine Learning

When considering the SVM-stride classifier and three neural network approaches (NN-stride, LSTM, CNN) for classifying subject groupings, both the SVM-stride and NN-stride, which are based on a parametric representation of strides, showed a higher prediction ability (Table 4) and higher AUROC values (Table 5) than the time series-based approaches (LSTM and CNN).

## 4. Discussion

With this study, we extended an existing investigation of the influence of environmental settings on gait patterns in NPH patients by comparing measurements taken in a controlled lab environment to those taken over 72 h in non-controlled real-world settings. We also tested the efficacy of each environment for providing the best diagnostic information by assessing the ability of data collected in each environment to discriminate NPH patients from an age-matched control group based on gait features. Lastly, we introduced SVM and neural network classifiers in order to analyze individual stride profiles of patients affected by NPH and compared approaches based on the time series versus a parametric representation of strides.

### 4.1. Real-World versus Controlled Environment

The intra-group comparisons between gait patterns in the two environments for NPH patients revealed a significant difference in gait performance, which is in accordance with the trend in the control groups, notably a decrease in stride length and gait speed, as well as an increase in step width, outward rotation, stance/swing ratio, double support phase, and variability of the stride time. Interestingly, the lower cadence and the slower stride time observed in the real-world setting for EHC did not seem to translate onto NPH patients, for which these metrics remained invariant. Concurrently, stride length and gait speed both decreased in the real-world environment, with the former having a considerably lower level of significance for the EHC group (p=0.0935) compared to the NPH group (p=0.0073). Considering the strong relationship between gait speed, stride length and stride time, we may postulate that whilst both subject groups aim at walking with a higher speed in controlled lab settings, EHC subjects achieve this through a faster cadence, whereas NPH subjects seem to focus on walking with longer strides. Subjects from the NPH group also show a significantly lower foot clearance in the real-world environment, commonly associated with neurological motor disorders such as Parkinson’s disease or stroke-related physical deficits [22,23,24].

Given that for the majority of the gait parameters, the environment-driven difference shows a high significance for EHC and NPH as opposed to the younger subject group, the assumption that elderly subjects are particularly influenced by the environment and the apprehension caused by a supervising observer in a lab setting [4,25] is to be extended to NPH patients, with a potential generalization to other neurological motor disorders.

### 4.2. Elderly Subjects versus NPH Patients

The second part of our statistical analysis evaluated the ability to discriminate between elderly subjects and NPH patients with respect to both real-world and controlled environments, firstly using the Mann–Whitney U-test and secondly using a logistic regression classifier. Measurements taken in real-world environments seem to produce larger inter-group differences than measurements taken in the lab (Table 2). Here, most of the gait parameters showed a considerable inter-group difference (with the exception of the outward rotation and the step width), but the comparison conducted in the real-world setting was characterized by a much higher confidence, with most parameters associated with a lower *p*-value in the real-world setting. The ability to better discriminate NPH subjects from EHC in the real-world environment was further tested through a logistic regression classifier that considers the interplay between the median values of the gait parameters and builds subject-specific profiles. The confusion matrices (Table 3) show a clear and consistent improvement in the classification ability when considering the real-world measurements, and the outcome of the overall investigation therefore supports the use of home-monitoring systems for cases of neurological diseases that manifest gait disorders, as for example investigated by studies on Parkinson’s disease [25]: in addition to further proving the influence of normal pressure hydrocephalus on the patient walking patterns compared to healthy subjects, these results also propose home-monitoring to be an incisive method and better diagnostic tool for the identification of symptoms than measurements that take place in laboratory settings.

Whilst this system has been proven to be effectively discreet during extended testing periods, it requires an initial calibration process that makes the setup cumbersome for both the clinician and patient. The calibration process is specifically needed for the estimation of the step width and the foot’s outward rotation during ground contact; however, these two parameters do not seem to discriminate between EHC and NPH patient groups. This could be due to the relatively high error in step width estimation [4] and the high sensitivity of the outward rotation parameter to the sensor placement and potential misalignment. The difficulty and inaccuracy in estimating these parameters via a wearable device, combined with the cumbersome nature of the required calibration process and the low significance of the inter-group differentiation of EHC and NPH, reduce the importance of these parameters. Although both outward rotation and step width have been indicated as relevant gait features expressly for NPH patients [5], the current results indicate that inter-group differentiation can successfully occur without taking them into consideration, and therefore, the current gait analysis protocol can be streamlined by eliminating the initial calibration process. Such improvements in efficiency make the current gait analysis system far easier to integrate as a tool for supporting clinical decision-making in the diagnosis of NPH, since the measurements could potentially start in the clinician’s office after attaching the sensors to the patient. It must be noted, however, that outward rotation and step width have not been discredited in the context of NPH diagnosis, and further development of the attitude estimation algorithms of the IMUs will aim to produce a more accurate estimation of these two parameters, which could still provide an improvement for the inter-group differentiation.

### 4.3. Stride Classification through Machine Learning

**Figure 4:**
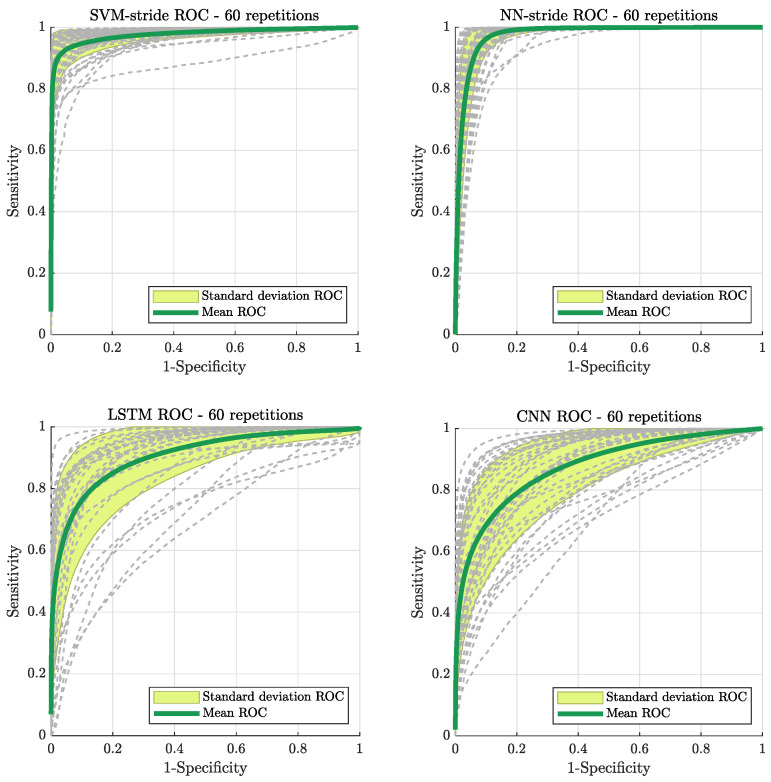
The four charts illustrate the receiver operating characteristic (ROC) curves computed for every model and averaged over 60 cross-validation repetitions (mean curve indicated with thick green line). The curves represent the pairs of sensitivity and specificity of the classifier when trialing all possible prediction thresholds. An ideal classifier would correspond to a pair of maximum specificity and maximum sensitivity of 1, hence a clustering towards the upper left corner is desired.

In the last part of the study, each subject’s strides from the EHC and NPH groups were considered individually, and different machine learning models (SVM-stride, NN-stride, LSTM, CNN) were trained to recognize class-specific features and correctly classify strides as belonging to either an EHC subject or NPH patient. Here, a perfect classification would have resulted in a 100% correct detection rate of the true class (c.f. Table 4). Amongst the four stride classifiers that were implemented, approaches based on the parametric representation of the strides (SVM-stride and NN-stride) resulted in better CDRs overall. With the exception of a couple of subjects per group, the percentages of correctly classified strides lie above 90%. As expected, elderly healthy subjects that performed relatively poorly compared to the group average (e.g., EHC 4, EHC 7) had lower CDR, with the same happening for NPH patients that displayed above-average gait performance (e.g., NPH 2, NPH4, NPH 6). Considering the completely random seeding during the Monte Carlo cross validation process, the similar subject-specific outcome of these two methods is a good indication that the interplay between gait parameters is successfully and consistently captured during the training process. Comparing the ROC curves of all four classifiers (shown in Figure 4) confirm the better performance of the SVM-stride and NN-stride models on this metric as well. The two parameter-based classifiers are associated with higher AUROC values and smaller variance across the cross-validation repetitions, which gives an important indication of the generalizability of the classifiers.

The time series-based approaches (LSTM and CNN) use the kinematic data directly to represent each stride and performs the classification either by learning to predict the shape of the time series-based on long and short-term dependencies within the time sequence or by learning to distinguish the time series’ specific visual features after encoding the signal into images. These two approaches had a comparable outcome, with similar AUROC values and with relatively good CDR for EHC class strides but considerably worse predictions for NPH strides. Since the size of the filters and layers within the deep neural networks have been only partially optimized, one explanation could be that gait patterns specific to NPH subjects are still not fully identified, and therefore, the classification skews towards more false EHC predictions. Because of the different groups’ size, and given that EHC subjects took on average three times more strides during the 72 h of measurement, it could also be hypothesized that the high false EHC prediction rate for NPH strides could be due to the entire data-set being slightly skewed. This issue could be addressed by implementing oversampling of the minority data, an effective and robust counter-measure to imbalanced data [21].

The predictions of the four stride classifiers showed that representing the strides with gait parameters (as in the case for the SVM-stride and NN-stride models) seems to yield the best overall accuracy and AUROC values. This could be due to the fact that the gait parameters used as predictor variables have been defined according to prior knowledge concerning walking features that have been clinically studied for years, and therefore the parametric representation of the strides could be more effective in encompassing the whole dynamic of the gait cycle and consequently better at describing gait patterns. In other words, the chosen set of gait parameters is, in fact, the outcome of an extensive and optimized feature selection process. The time series-based models (LSTM and CNN) were trained to learn class-specific features from scratch, but nevertheless performed comparably to SVM-stride and NN-stride. It is important to note that these methods were only subjected to a partial optimization of the respective hyper-parameters, and therefore, the derived assessments could suffer a loss of generality.

## 5. Conclusions

The results of our study demonstrate that NPH patients exhibit significantly different walking patterns in real-world environments compared to lab settings, hence supporting the idea of utilising home monitoring systems for subjects with indications of gait disorders. Since external rotation and step width did not contribute to the inter-group differentiation, it seems that the initial calibration process may not be necessary, hence reducing the hurdles for integrating the current gait analysis system into current assessment protocols. Finally, the four stride classifiers proposed showed a consistent prediction behavior, but with SVM-stride and NN-stride classifiers both outperforming the time series-based classifiers (LSTM and CNN). However, further optimization of the relevant LSTM and CNN hyper-parameters looks promising for better characterization of stride types and identifying new class-specific features. The approaches presented here therefore provide a clear basis for supporting clinical decision-making in the identifications of gait disorders related to NPH.

## Figures and Tables

**Table 1:** Resulting relative differences and *p*-values from Wilcoxon signed rank test comparing the gait parameters of all three subject groups in controlled and real-world environments. Significant results are indicated in bold, with blue representing values closer to 0 and white representing values closer to 1.

No	Parameter	Unit	YHC—20 Subjects		EHC—20 Subjects		NPH—12 Subjects	
			Rel. Diff.	Wilcoxon	Rel. Diff.	Wilcoxon	Rel. Diff.	Wilcoxon
1	Stride length	m	−0.9%	0.7333	−5.6%	0.0935	−43.4%	**0.0073**
2	Sensor clearance	m	0.3%	0.8078	0.0%	0.8496	−24.9%	**0.0117**
3	Gait speed	m/s	−2.1%	0.7990	−12.1%	**0.0068**	−44.3%	**0.0073**
4	Outward rotation	rad	19.4%	**0.0094**	15.8%	**0.0018**	16.6%	**0.0317**
5	Step width	m	31.5%	**0.0010**	−17.7%	**0.0046**	23.4%	**0.0293**
6	Hand distance	m	5.7%	0.1737	3.6%	0.5377	−6.8%	0.4370
7	Hand max. ang. velocity	rad/s	7.3%	0.0868	−1.3%	0.8373	−16.4%	0.6107
8	Number of steps per $180^{\circ }$	steps	19.3%	**0.008**	19.8%	**0.0016**	6.3%	0.3276
9	Stance phase	%	0.9%	0.1982	0.6%	0.5285	4.0%	**0.0073**
10	Swing phase	%	−1.5%	0.3749	−0.8%	0.5606	−7.6%	**0.0209**
11	Stance/swing ratio	%	3.0%	0.0868	2.5%	0.5182	9.4%	**0.0098**
12	Double support phase	%	6.8%	0.0560	5.1%	0.0926	16.8%	**0.0183**
13	Cadence	steps/min	−0.12%	0.9477	−6.0%	**0.0056**	−0.3%	0.7339
14	Stride time	s	−0.6%	0.8414	4.8%	**0.0058**	−1.28%	0.6906
15	Variability stride time	%	51.3%	**0.0013**	57.6%	**0.0013**	−43.41%	**0.0073**

**Table 2:** Results from the Mann–Whitney U-test comparing the gait parameters of normal pressure hydrocephalus (NPH) patients and the healthy elderly control group (EHC). Significant results are indicated in bold, with blue representing values closer to 0 and white representing values closer to 1.

No	Parameter	Unit	Controlled—*p*-Value	Non-Controlled—*p*-Value
1	Stride length	m	**<0.0001**	**<0.0001**
2	Sensor clearance	m	**0.0005**	**<0.0001**
3	Gait speed	m/s	**<0.0001**	**<0.0001**
4	Outward rotation	rad	0.3811	0.3023
5	Step width	m	0.3603	0.4249
6	Hand distance	m	**0.0068**	**<0.0001**
7	Hand max. ang. velocity	rad/s	**0.0003**	**<0.0001**
8	Number of steps per $180^{\circ }$	steps	**<0.0001**	**0.0001**
9	Stance phase	%	0.2200	**0.0006**
10	Swing phase	%	**0.0409 **	**0.0002**
11	Stance/swing ratio	%	0.0703	**0.0003**
12	Double support phase	%	0.1554	**0.0006**
13	Cadence	steps/min	0.0589	0.2739
14	Walk-cycle time	(s)	0.0562	0.1913
15	Variability walk-cycle time	%	**0.0029**	0.2201
16	Variability stride length	%	**0.0005**	**0.0004**
17	Variability stance/swing ratio	%	**0.0001**	**0.0002**

**Table 3:** Logistic regression classifier prediction in the controlled environment (left) and in the real-world environment (right) for the elderly healthy control group (EHC) and normal pressure hydrocephalus patients group (NPH). Green represents values closer to 100%, and white indicates values closer to 0%.

**Controlled environment**				**Real-world environment**			
True Class	EHC	89.5%	10.5%	True Class	EHC	95.8%	4.2%
	NPH	24.4%	75.6%		NPH	8.4%	91.6%
		EHC	NPH			EHC	NPH
		Predicted Class				Predicted Class	

**Table 4:** The table displays the percentages of strides classified by the four classifiers: support vector machine (SVM-stride), feedforward neural network (NN-stride), long short-term memory neural network (LSTM) and convolutional neural network (CNN). Using at least 20 iterations for the Monte Carlo cross-validation, all subjects are considered in the test set at least once. Green represents values closer to 100%, and white indicates values closer to 0% match.

True Class	Subject	SVM Class Prediction		NN Class Prediction		LSTM Class Prediction		CNN Class Prediction	
		EHC	NPH	EHC	NPH	EHC	NPH	EHC	NPH
EHC	1	96.2%	3.8%	96.3%	3.7%	87.6%	12.4%	60.5%	39.4%
	2	98.1%	1.9%	97.7%	2.3%	81.0%	19.0%	97.0%	2.9%
	3	90.0%	10.0%	89.1%	10.9%	79.5%	20.5%	88.7%	11.2%
	4	67.3%	32.7%	54.6%	45.4%	97.0%	3.0%	82.8%	17.1%
	5	96.6%	3.4%	96.4%	3.6%	59.0%	41.0%	94.0%	5.9%
	6	92.0%	8.0%	92.1%	7.9%	62.7%	37.3%	70.0%	29.9%
	7	66.0%	34.0%	43.0%	57.0%	90.6%	9.4%	74.6%	25.3%
	8	97.4%	2.6%	97.7%	2.3%	98.2%	1.8%	95.4%	4.5%
	9	98.3%	1.7%	98.6%	1.4%	97.7%	2.3%	95.5%	4.4%
	10	98.9%	1.1%	98.6%	1.5%	99.4%	0.6%	97.4%	2.5%
	11	98.8%	1.2%	99.0%	1.0%	98.9%	1.1%	99.2%	0.7%
	12	98.9%	1.1%	98.8%	1.2%	86.3%	13.7%	94.9%	5.0%
	13	98.4%	1.6%	98.8%	1.2%	97.9%	2.1%	98.6%	1.3%
	14	97.5%	2.5%	99.0%	1.0%	99.3%	0.7%	98.2%	1.7%
	15	98.3%	1.7%	98.0%	2.0%	88.9%	11.1%	91.4%	8.5%
	16	95.2%	4.8%	94.5%	5.5%	95.0%	5.0%	96.0%	3.9%
	17	95.9%	4.1%	96.1%	3.9%	92.3%	7.7%	90.2%	9.7%
	18	99.1%	0.9%	99.3%	0.7%	98.8%	1.2%	98.1%	1.8%
	19	99.1%	0.9%	99.9%	0.1%	96.2%	3.8%	97.6%	2.3%
	20	95.7%	4.3%	98.0%	2.0%	99.6%	0.4%	98.8%	1.1%
NPH	1	11.0%	89.0%	10.4%	89.6%	45.3%	54.7%	57.2%	42.7%
	2	60.4%	39.6%	51.5%	48.5%	54.0%	45.9%	74.7%	25.2%
	3	6.8%	93.2%	21.9%	78.1%	28.7%	71.3%	49.8%	50.1%
	4	29.7%	70.3%	7.4%	92.6%	13.6%	86.4%	44.1%	55.8%
	5	2.2%	97.8%	3.8%	96.2%	13.3%	86.7%	16.8%	83.1%
	6	61.7%	38.3%	63.1%	36.9%	76.4%	23.6%	85.2%	14.7%
	7	1.6%	98.4%	11.7%	88.3%	9.5%	90.5%	47.2%	52.7%
	8	12.8%	87.2%	34.9%	65.1%	44.2%	55.8%	52.1%	47.8%
	9	1.9%	98.1%	2.6%	97.4%	32.9%	67.1%	34.0%	65.9%
	10	1.0%	99.0%	0.8%	99.2%	10.1%	89.9%	11.0%	88.9%
	11	1.1%	98.9%	1.8%	98.2%	3.4%	96.6%	6.5%	93.4%
	12	9.9%	90.1%	18.6%	81.4%	19.5%	80.5%	25.9%	74.0%

**Table 5:** The table lists the area under the receiver operating characteristic curves (AUROC) for all four classifiers: support vector machine (SVM-stride), feedforward neural network (NN-stride), long short-term memory neural network (LSTM) and convolutional neural network (CNN). Mean AUROC values and standard deviation (std) values have been calculated over 60 cross-validation repetitions.

Method	Classifier	Mean AUROC	std AUROC	Accuracy
Parameter representation	SVM-stride	0.976	0.020	89.9
	NN-stride	0.976	0.016	88.0
Time-series representation	LSTM	0.903	0.076	83.0
	CNN	0.876	0.084	78.5

## Data Availability

Raw data from this study are not publicly available due to ethical constraints where test subjects were guaranteed that data would be used only anonymously and not passed to external users. Requests to access the datasets under specific conditions should be directed to M.S.D., marischm@ethz.ch”.
